# A rare case of a completely thrombosed bilobed giant intracranial aneurysm of the anterior cerebral artery with spontaneous parent vessel thrombosis: case report

**DOI:** 10.1186/s12883-019-1529-6

**Published:** 2019-11-23

**Authors:** Mehdi Chihi, Ramazan Jabbarli, Oliver Gembruch, Sarah Teuber-Hanselmann, Marvin Darkwah Oppong, Daniela Pierscianek, Alexander Radbruch, Martin Glas, Mark Stettner, Ulrich Sure

**Affiliations:** 1 Department of Neurosurgery, University Hospital Essen, University of Duisburg-Essen, Essen, Germany; 2 Institute of Neuropathology, University Hospital Essen, University of Duisburg-Essen, Essen, Germany; 3 Institute of Diagnostic and Interventional Radiology and Neuroradiology, University Hospital Essen, University of Duisburg-Essen, Essen, Germany; 4 Department of Neurology, University Hospital Essen, University of Duisburg-Essen, Essen, Germany

**Keywords:** Giant intracranial aneurysm, Intraluminal thrombosis, Clipping, Parent vessel

## Abstract

**Background:**

A huge spherical intracranial mass can sometimes be misdiagnosed, due to the lack of typical radiographic features. Thrombosed giant intracranial aneurysms (GIAs) are an uncommon but still a possible differential diagnosis that must be kept in mind to guarantee the best surgical approach and resection of the lesion. We describe an extremely rare case of a huge bifrontal mass mimicking a cystic echinococcosis, in which the surgery unveiled a completely thrombosed GIA of the left anterior cerebral artery (ACA).

**Case presentation:**

A 61-year-old patient complained about intermittent weakness of the right leg, mild holocephalic headache, beginning cognitive deficits and lethargy. Magnetic resonance imaging (MRI) showed a huge partially calcified and bilobed frontal mass with peripheral edema. Based on a time-resolved angiography with interleaved Stochastic trajectories MRI (TWIST-MRI), a vascular origin of the lesion was considered unlikely. Therefore, the surgery was performed under the suspicion of a cystic echinococcosis but revealed a bilobed GIA of the left ACA with a parent vessel thrombosis. Although only a limited left frontal craniotomy was performed, a proximal control of the parent vessel could be ensured, and the aneurysm was successfully clipped. The patient showed postoperatively no new neurological deficits.

**Conclusions:**

Completely thrombosed GIAs with parent vessel thrombosis are rare lesions that might be misdiagnosed if typical radiographic features are missing. Thus, in case of an intracranial spherical mass with signs of intralesional hemorrhage and mural calcifications, presence of a completely thrombosed GIA should be considered as a possible differential diagnosis.

## Background

Giant intracranial aneurysms (GIAs) are rare and heterogeneous lesions with complex vascular anatomy [1] and represent almost 5% of intracranial aneurysms (IAs) [2]. They typically become symptomatic between the 4th and the 7th decade, with a female to male ratio ranging from 1:1 to 3:1 [2]. The incidence of intraluminal thrombosis ranges from 10 to 30% of the cases, [3] however, a complete aneurysmal thrombosis is extremely rare [4]. The majority of these aneurysms are located in the internal carotid artery (ICA) and in the middle cerebral artery (MCA), the anterior cerebral artery (ACA) being an exceptional location [1].

In this report, we present an unusual case of a completely thrombosed GIA of the left ACA, that was preoperatively misdiagnosed because of the lack of typical radiographic features.

## Case presentation

A 61-year-old male patient was referred to our neurosurgical department with a 10-day history of an intermittent weakness of the right leg, mild holocephalic headache, beginning cognitive deficits and lethargy. A recent stay in America, China and East Africa was reported. There was no fever and no weight loss. Physical examination showed an ataxic gait. During the hospital stay, the patient developed a focal seizure of the right leg, which was treated with levetiracetam.

### Radiological findings(Fig. 1)

Magnetic resonance imaging (MRI) obtained from the referring hospital reported two well-defined frontal bihemispheric masses, 54 and 40 mm in diameter, with a slight perifocal edema. The patient was admitted to the intermediate care unit and a computed tomography angiography was performed. The latter showed two large lesions in both frontal regions, partly inhomogeneous, with central hyperdense parts and with partial mural calcifications. A thorax and abdomen CT showed no tumor manifestation. A time-of-flight MRI (TOF-MRI) demonstrated the right ACA shifted to the lateral wall of the lesion, whereas only the proximal left ACA (A1-segment) was displayed. Time-resolved angiography with interleaved stochastic trajectories MRI (TWIST-MRI) was also performed and showed neither contrast enhancement nor intraluminal filling of both masses.

**Fig. 1:**
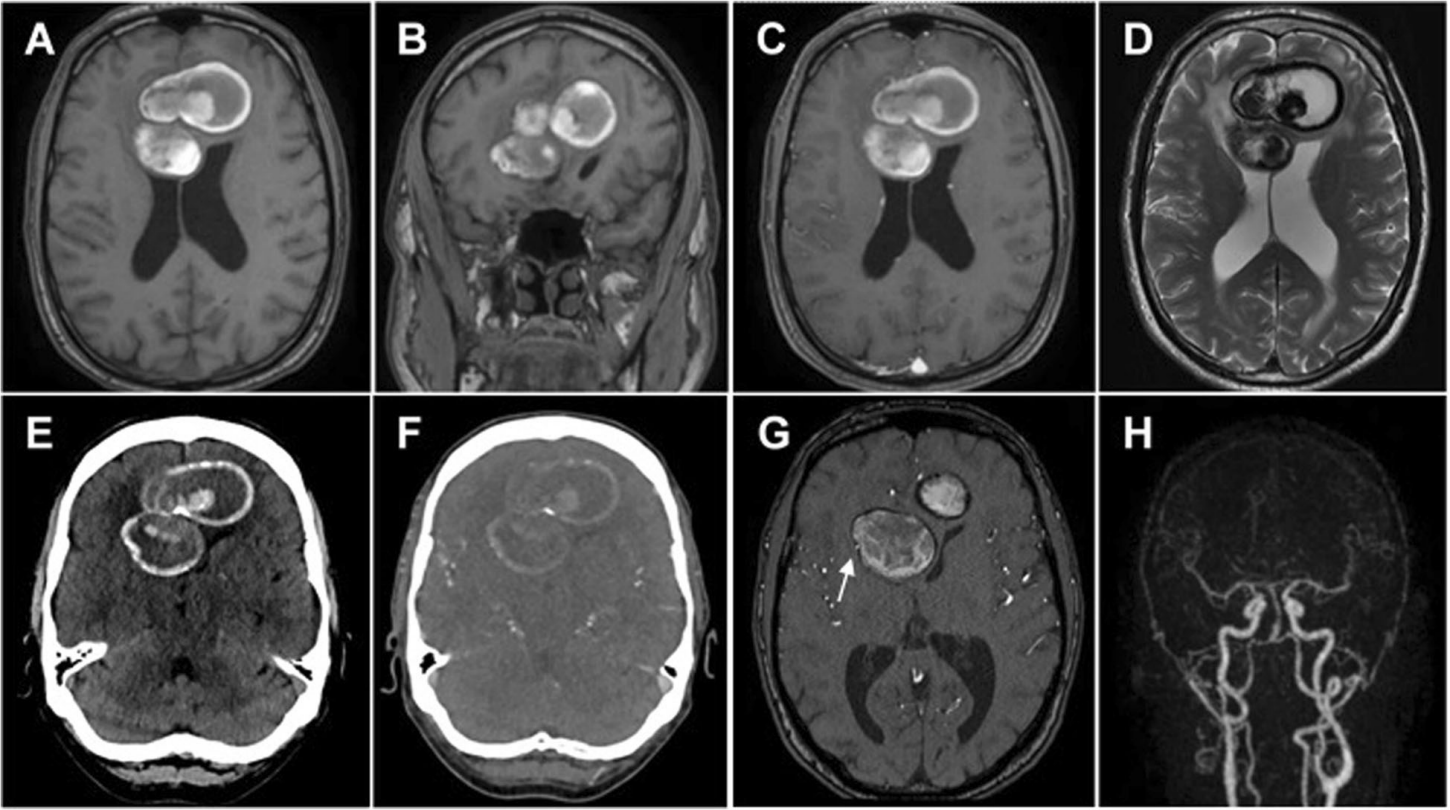
Preoperative imaging. Preoperative imaging showing in the cranial MRI a bilobed and bihemispheric huge mass (54 and 40 mm in diameter) of both frontal lobes with a crescent shaped hyperintensity of the lesion wall in the T1-weighted frame (**a**, **b**), without rim enhancement of the lesion wall or intraluminal flow void after administration of Gadolinium (**c**) and with perifocal edema in the T2-weighted frame (**d**). Non-enhanced cranial CT demonstrated a partially hyperdense mass lesion with partial peripheral and intralesional calcifications as well as intramural hemorrhage and slight perifocal edema (**e**), but without contrast enhancing ring after contrast administration (**f**). TOF-MRI (**g**) showed the right ACA at the lateral wall of the lesion (white arrow). TWIST-MRI (**h**) demonstrated neither contrast enhancement nor intraluminal filling of both masses. MRI: Magnetic resonance imaging, CT: Computed tomography, TOF: Time-of-flight, ACA: Anterior cerebral artery, TWIST-MRI: Time-resolved angiography with interleaved stochastic trajectories MRI

### Laboratory findings

Blood and cerebrospinal fluid (CSF) screening presented no signs of infection. There were no tumor cells in the CSF. Parasitological examination of blood and CSF was performed. A low antibody concentration against Echinococcus multilocularis was detected in the patient’s CSF (1:20).

### Surgical treatment (Fig. 2)

Because of the recent foreign stay in East Africa and the above-mentioned CSF results, the diagnosis of a cerebral cystic echinococcosis was suspected. Consequently, no digital subtraction angiography (DSA) was performed, as a GIA in this case was besides radiologically considered extremely unlikely, and a microsurgical resection of the lesion was indicated. Thus, a left frontal craniotomy was performed. The preparation showed a very hard cystic lesion with vasa vasorum at the lesion wall evoking the differential diagnosis of a thrombosed aneurysm. The preparation of the GIA was most difficult due to the absence of a proximal control through the performed frontal craniotomy. After a careful and meticulous dissection, the lesion could be finally prepared in all directions. Furthermore, the aneurysmal parent vessel was found. The intraoperative Indocyanine green angiography demonstrated a very low blood flow in this thrombosed vessel. After clipping of the parent vessel, the two aneurysmal sacs were opened, and the thrombi and calcifications were resected.

**Fig. 2:**
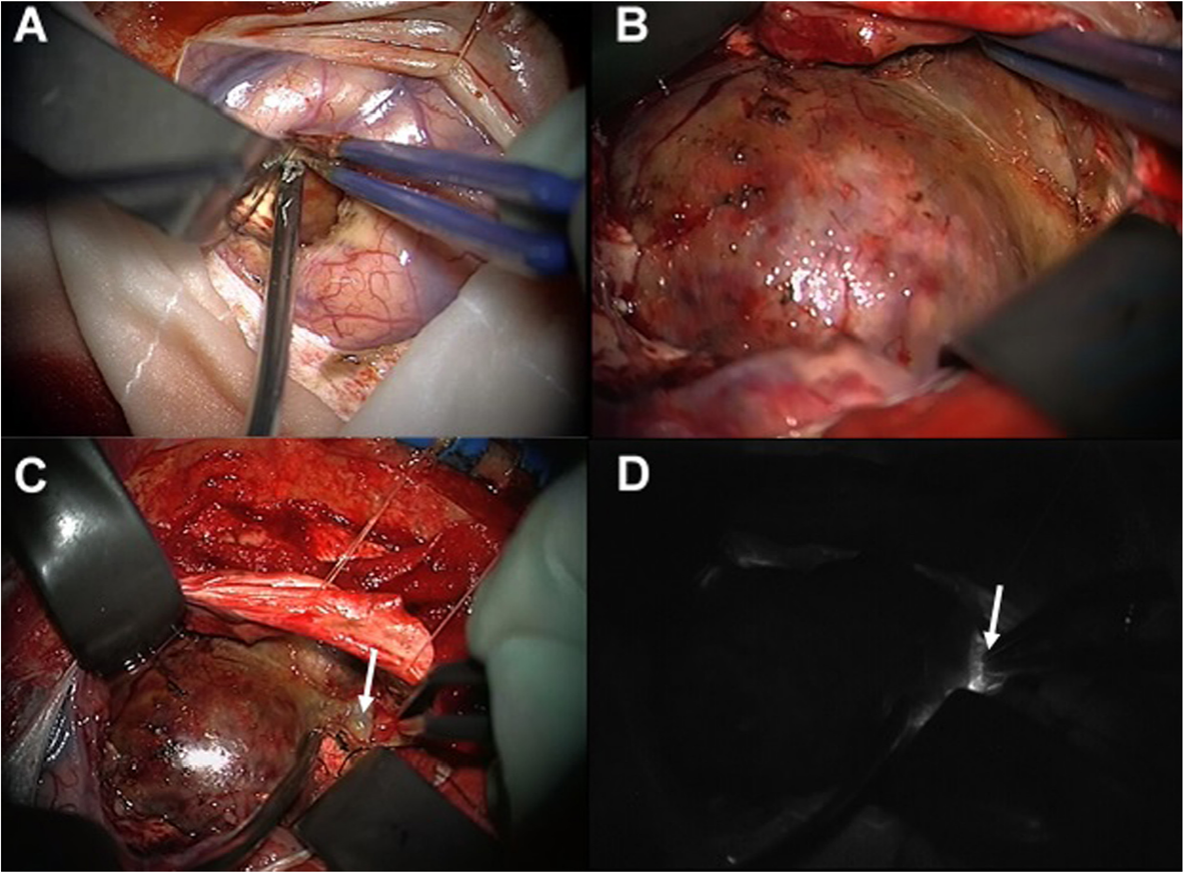
Intraoperative photographs. After left frontal craniotomy and corticotomy, a hard-cystic lesion was found (**a**) with vasa vasorum at its wall (**b**). Meticulous dissection in all direction was performed until the parent vessel was found (**c**, white arrow). The Indocyanine green angiography showed only a very low blood flow in the parent artery (**d**, white arrow)

### Histopathology (Fig. 3)

Histopathological examination revealed wall fragments of an artery of large caliber adjacent to thrombotic material. The vessel wall was irregularly thickened with eccentric fibrosis or abnormal wall thinning; an internal elastic lamina was seen only focally. Hemosiderin deposits, calcifications and inflammatory infiltrates were detectable within the remnants of the vessel wall.

**Fig. 3:**
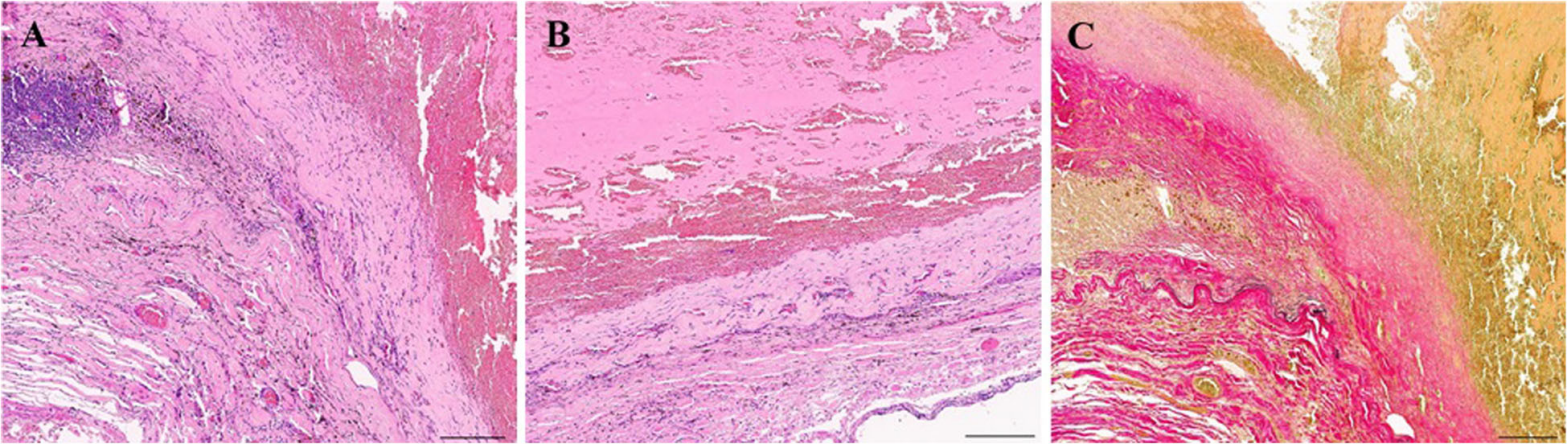
Histopathological findings. Hematoxylin-eosin staining revealed wall fragments of a large caliber artery with eccentric fibrosis, inflammatory infiltrates and hemosiderin deposits (**a**) or abnormal wall thinning (**b**) adjacent to thrombotic material. The internal elastic lamina ends abruptly (Elastica-van-Gieson staining; **c** Scale bar: 200 μm)

### Postoperative course and follow-up (Fig. 4)

Postoperatively, the patient did well and had no neurological deficits. A DSA showed no aneurysmal remnant after clipping. A collateral circulation coming from both MCAs and the posterior circulation was detected. At the 6-month follow-up, the patient recovered from the operation and could walk without assistance.

**Fig. 4:**
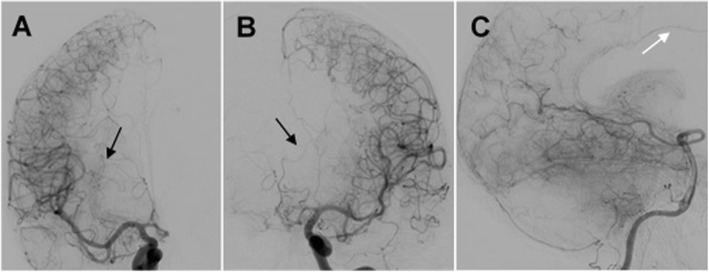
Postoperative imaging. Postoperative DSA showing no aneurysmal remnant after clipping the bilobed GIA of the anterior circulation. A collateral circulation coming from both MCAs (**a** and **b**, black arrows) and the posterior circulation (white arrow) was detected (**c**). DSA: Digital substraction angiography, GIA: Giant intracranial aneurysm, MCA: Middle cerebral artery

## Discussion

The natural history of GIAs is featured with three pathological events: spontaneous thrombosis, growth leading to a mass effect and rupture causing a subarachnoid hemorrhage [2]. These are characterized by a high mortality rate, essentially for aneurysms located on the posterior circulation, [5] as the rupture rate in 5 years ranges between 40 and 50% [6]. Most of the time, as in our case, GIAs are diagnosed in the course of a pseudotumoral syndrome, where the mass effect of the IA leads to a gradual neurologic deterioration [7].

Complete intraluminal thrombosis is uncommon and occurs especially in large and giant cerebral aneurysms in 13–20% of cases [4]. This phenomenon is explained by endothelial damage due to hemodynamic stress on the aneurysmal wall [4]. CT characteristics of thrombosed IAs are peripheral ring enhancement, curvilinear mural calcification, intraluminal mixed high-density calcification and the presence of “target sign” [8]. On the other hand, MRI features include “onion skin” appearance, luminal flow void, luminal enhancement and perianeurysmal edema on T2-weighted sequences [9]. In our case, neither intraluminal flow void nor peripheral contrast enhancement was detected on MRI and CT. Although all radiological examinations −excluding DSA− were carried out, a misdiagnosis was made because of the lack of typical radiographic features.

Moreover, the left A2-segment was not detected on the TOF-MRI. Based on this MRI feature and on the intraoperative fact that the parent vessel was probably coming from the left ACA, spontaneous occlusion of the parent artery (left A2-segment) harboring the GIA was suspected. Indeed, although this scenario is extremely rare, [4] it would probably explain the absence of intraluminal flow void and absence of enhancement of the aneurysmal parent vessel on both TOF- and TWIST-MRI. In fact, the mechanism of the spontaneous thrombosis of an aneurysm and its parent artery is not known [4]. Theories of local stretching, compression and distortion of the ICA could explain the occurrence of this complication in IAs of the cavernous segment, due to the presence of dural folds and bony structures at this location [10]. In our case, compression of the parent vessel by a growing partially thrombosed aneurysm, or retrograde propagation of the thrombus, [11] leading to an acute parent artery thrombosis and subsequently to a complete thrombosis of the aneurysm, are two possible mechanisms.

In this way, because of the limits of imaging, in front of a huge intracranial mass presenting with mural calcification and signs of hemorrhage, a completely thrombosed GIA must be considered as a differential diagnosis and must not be ruled out until the operation is performed. Preoperatively, a DSA is always helpful and must be included in the preoperative workup to visualize the parent vessel of the IA and plan the operation. Even though, in the case of a completely thrombosed aneurysm, DSA could be negative [12]. Furthermore, a well-trained vascular neurosurgeon must be available on the day of operation, especially in non-high volume vascular centers. In our case, although the diagnosis of GIA was made intraoperatively, careful and meticulous preparation of the aneurysm allowed us to find the parent artery and have a proximal control before clipping the aneurysm.

## Conclusions

Completely thrombosed GIAs with parent vessel thrombosis are rare lesions that might be misdiagnosed if typical radiographic features are missing. Thus, in case of an intracranial spherical mass with signs of intralesional hemorrhage and mural calcifications, presence of a completely thrombosed GIA should be considered as a possible differential diagnosis.

## Data Availability

All data generated or analyzed during this study are included in this published article.

## Abbreviations

ACA: Anterior cerebral artery
CSF: Cerebrospinal fluid
CT: Computer tomography
DSA: Digital subtraction angiography
GIA: Giant intracranial aneurysm
IA: Intracranial aneurysm
ICA: Internal carotid artery
MCA: Middle cerebral artery
MRI: Magnetic resonance imaging
TOF-MRI: Time-of-flight MRI
TWIST-MRI: Time-resolved angiography with interleaved stochastic trajectories MRI
